# A Modified Embedded-Atom Method Potential for a Quaternary Fe-Cr-Si-Mo Solid Solution Alloy

**DOI:** 10.3390/ma16072825

**Published:** 2023-04-01

**Authors:** Shiddartha Paul, Daniel Schwen, Michael P. Short, Kasra Momeni

**Affiliations:** 1 Department of Mechanical Engineering, University of Alabama, Tuscaloosa, AL 35487, USA; 2 Department of Mechanical Science and Engineering, University of Illinois at Urbana-Champaign, Urbana, IL 61801, USA; 3 Department of Computational Mechanics and Materials, Idaho National Laboratory, Idaho Falls, ID 83402, USA; 4 Department of Nuclear Science & Engineering, Massachusetts Institute of Technology, Cambridge, MA 02139, USA

**Keywords:** MEAM, nuclear fuel materials, molecular dynamics, alloy development

## Abstract

Ferritic-martensitic steels, such as T91, are candidate materials for high-temperature applications, including superheaters, heat exchangers, and advanced nuclear reactors. Considering these alloys’ wide applications, an atomistic understanding of the underlying mechanisms responsible for their excellent mechano-chemical properties is crucial. Here, we developed a modified embedded-atom method (MEAM) potential for the Fe-Cr-Si-Mo quaternary alloy system—i.e., four major elements of T91—using a multi-objective optimization approach to fit thermomechanical properties reported using density functional theory (DFT) calculations and experimental measurements. Elastic constants calculated using the proposed potential for binary interactions agreed well with ab initio calculations. Furthermore, the computed thermal expansion and self-diffusion coefficients employing this potential are in good agreement with other studies. This potential will offer insightful atomistic knowledge to design alloys for use in harsh environments.

## 1. Introduction

As interest in nuclear energy grows, developing durable materials for advanced fission and fusion reactors has become a major priority [1]. The expected lifetime of Generation IV reactors is more than 60 years, with operating temperatures in the range of 1000 K [2]. These reactors require materials that can sustain higher temperatures and radiation fluxes than the existing ones [3]. Radiation-induced hardening and embrittlement are a concern as they can cause materials used in these applications to fail and hence compromise the reliability of reactors [4]. Ongoing research aims to enhance the safety of existing nuclear power plants, design advanced next-generation reactors, and develop accident-tolerant fuel (ATF) with improved fuel and cladding concepts [5]. For example, one of the goals of the ATF fuel concepts is to replace zirconium alloy fuel cladding with Fe-Cr-based alloys due to their higher oxidation resistance [6,7].

Quaternary alloy systems have garnered significant interest in recent years due to their unique properties, versatility, and potential for new material discovery. For example, researchers have shown that the addition of a fourth element can enhance the mechanical strength and thermal stability of the material, making it suitable for high-temperature applications [8]. Additionally, quaternary alloys have been shown to possess improved corrosion resistance and electrical conductivity [9]. Moreover, the tunability of quaternary alloys has led to the discovery of novel materials with unique properties. For instance, researchers have recently discovered a quaternary alloy with a high hydrogen storage capacity, which has potential applications in hydrogen fuel cell technology [10,11]. The addition of a fourth element can stabilize the crystal structure, prevent defects and phase transformations, and improve the overall material stability. This increased stability is critical in harsh environments, such as high-temperature or high-pressure conditions [12]. Overall, the significance of quaternary alloy systems lies in their unique properties, versatility, and tunability.

There are numerous candidate materials for advanced nuclear reactors, out of which ferritic/martensitic steel (FMS) with 9–12% Cr, such as T91, is considered the most reliable due to its high thermal conductivity, low thermal expansion coefficient, high-temperature strength, and very low void swelling rate under neutron irradiation [13,14,15]. Cr in FMS strongly influences the radiation-induced microstructure after irradiation, e.g., the presence of 12 wt% Cr in steel provides defect trapping and suppression of radiation swelling [16]. The Cr concentration results in the segregation of defects, grain boundaries, and interfaces [17]. The introduction of Si can promote the formation of Cr-Si compounds, which enhances thermal stability and consequently improves high-temperature strength [18]. Adding Mo, together with Cr and Ni, prompts their corrosion resistance [19]. The chemical composition of T91 is Febal-C0.1-Cr8.87-Si0.2-Mo0.87-V0.20-Mn0.39 wt% [16,20,21], where Fe/Cr/Si/Mo are the four key elements. These alloys, combined with other metals in a functionally graded structure, form a multimetallic layered composite (MMLC) that displays higher radiation resistance than any of the individual components due to their existing interfaces. An interatomic potential that can capture the behavior of this quaternary alloy paves the way to design new materials for applications under extreme conditions. 

Efficient computational tools have been developed to perform simulations within timespans not accessible to experimental studies [22,23]. We may refer to large-scale continuum simulations [24,25,26,27,28,29,30,31,32,33,34,35,36,37,38,39,40,41,42,43,44,45,46,47], phase-field simulations for capturing the microstructure [48,49,50,51,52,53,54,55], MD simulations to capture the atomistic mechanisms [2,47,56,57,58,59,60,61,62,63,64,65,66,67], and multiscale simulations to capture the broad spectrum of materials and processes response [24,25,46,68,69,70,71,72,73]. Out of several computational methods, molecular dynamics simulation allows the capturing of materials evolution with atomistic accuracy, including the radiation damage mechanism. Molecular dynamics modeling approaches rely heavily on an interatomic potential that accurately represents the irradiated material’s physical characteristics [74]. 

Numerous semi-empirical potentials for metals and alloys have been created over time [75]. These potentials were subsequently expanded and modified to include electronic density, leading to many-body interatomic potential models considered to be the state of the art for atomistic simulations. The MEAM potential, which incorporates angular dependence and a 2NN formulism for metallic alloys, has been developed and can predict the phase stability of diverse alloys, including high-entropy alloys with numerous constituent elements during irradiation [76,77]. Despite these advances, developing MEAM potentials for ternary and quaternary multicomponent metallic liquids is particularly difficult.

Most atomic-scale simulations of multicomponent alloys focus on unary and binary constituents [78]. Few other studies have been conducted on the development of MEAM potentials beyond ternary alloys [77,79,80,81,82] and compute the structural and elastic properties in Fe-Cr-Si alloys and their intermetallic unary, binary, and ternary alloys [83]. However, we are unaware of any MEAM potential for the quaternary alloy Fe-Cr-Si-Mo that is considered in the present study. Although T91 contains other elements, as reported [16,20,21], our quaternary MEAM potential provides valuable insight for developing ATF cladding materials, materials for advanced nuclear reactors, and materials for application in extreme environments based on T91. We simulated ten unary and binary system interactions for the present model and fitted them to structural and energetic parameters.

Several experiments have shown that T91 (Fe-Cr-Mo) and Fe-Cr-Si alloys exhibit excellent localized corrosion resistance and irradiation resistance, even after prolonged exposure to high radiation levels [84,85]. However, accurate theoretical characterization of these alloys at the atomic level is still lacking, mainly due to a scarcity of precise potential. Although the development of quaternary molecular dynamics (MD) potentials has shown promising results in predicting the mechanical properties of high-entropy alloys (HEAs), the lack of suitable potentials for some of the constituent elements, such as Si and Mo, hinders the development of new HEAs with unique structural properties [86]. Thus, our study is to develop accurate potentials for this Fe-Cr-Si-Mo quaternary system that can describe the atomic interactions in these complex alloys and ultimately enable the design of a new alloy/composite, along with HEAs with tailored properties.

## 2. Materials and Methods

### 2.1. Potential Development and Fitting

Using the molecular dynamics simulation code LAMMPS [87], we created a quaternary system consisting of Fe-Cr-Si-Mo in two stages. The first stage involved Fe-Cr-Si, and the second stage involved the addition of Mo. We capitalized on unary and binary systems for this study using an embedded-atom method (EAM) potential [88] to properly reproduce Fe-Cr, Fe-Si, and Fe-Mo characteristics and later merge them. Metallic atoms were treated using the EAM potential, while non-metallic atoms and metal/non-metal atoms were handled using the Tersoff approach [89]. We selected four interatomic potentials, including one for the Fe-Cr system [90], which was updated with recent experimental high-temperature data and ab initio calculations. We initially attempted to use an improved Tersoff potential for the Si system [91], but it was ultimately discarded due to its inability to properly describe the binary compounds, which had to rely solely on the Ziegler–Biersack–Littmark (ZBL) universal potential method [92]. To maintain consistency with the EAM method, we used the modified embedded-atom method (MEAM) [93], which incorporates directional bonding and a self-implemented ZBL. This method is more accurate than EAM–ZBL and has previously yielded satisfactory results in irradiation simulations [94].

In a recent study [77], the MEAM approach was used to simulate the unary and binary interactions of Fe-C-Cr-Mo, as listed in Table 1. Our own simulations using this potential accurately reproduced the properties of the Mo unary system. However, we found the data for Fe and Cr to be highly inconsistent, and we cannot definitively state that our desired potential accurately reproduces the T91 system, as the concentration of Fe and Cr is higher than the other constituents in our model. Additionally, no potential can completely describe the binary interactions of the Fe-Cr-Si-Mo system. Thus, we integrated five different potentials to fully describe the interactions in our quaternary system, complemented with a fitting/refining for accurate results and desired reproducible properties. Therefore, we selected and merged potential-containing unary and binary systems mentioned for each reference in Table 1. We have ten unary and binary system interactions, which are tested and fitted to reproduce basic structural and energetic parameters. Thus, we calibrated our designed potential to validate unary and binary systems’ applicability.

### 2.2. Unary Potential

For the mechanical property calculations of the unary components and potential validation, we evaluated the unit cell of each element. In its ground state, Fe, Cr, and Mo each adopt body-centered cubic (bcc) units, while Si has a diamond structure. We minimized the structure for each model and compared the resulting parameters with those in Table 2. Our calculations agree with MEAM, DFT, and experimental results and reproduce those parameters. Nevertheless, we detected some inconsistencies in the Fe system. As a result, we revised the potential parameters for the Fe system using [81]. 

### 2.3. Binary Potential

In terms of the unoptimized MEAM Potential (MEAM-A), in total, six binary interactions (Fe-Cr, Fe-Mo, Fe-Si, Cr-Mo, Cr-Si, and Si-Mo) represent the complete quaternary system. The potentials were tested and validated, without optimizing parameters for the quaternary systems, on their identified ground state structures of binary phase diagrams, at low temperatures for proper comparison. Table 3 presents the findings for the interactions that determine the structures examined for each system. The columns are divided as follows: cohesive energy (E_c_) in (eV), lattice parameter (a_lat_) in (Å); the elastic constants (C11, C12, and C44), shear modulus (G), Young modulus (E) and bulk modulus (B) in (GPa); and the unit less Poisson’s ratio (v). The computed results of this study are shaded. Initial and screening parameters are mentioned in the Supplementary Information.

For the Fe-Cr interaction, C12 and C44 deviate from the reference but maintain the overall relationship, which is reflected in the rest of the parameters that agree with the DFT calculations [100]. The initial parameters and variable settings for the Cr-Si system are presented in Supplementary Information. The Fe-Si interaction was measured for three different structures, the B20—known as the ground state—and a hypothetical B2 and B1 structure, to compare them with the available references [82,100]. The cubic crystal’s elastic properties and bulk modulus are calculated by Voigt–Reuss–Hill approximation [109] for Fe-Si B20, Cr-Mo B2, and Cr-Si P213 symmetry along axes such that C11=C22=C33, C12=C21=C23=C32=C13=C31, and C44=C55=C66. The off-diagonal shear components are zero, giving C45=C54=C56=C65=C46=C64=0; mixed compression/shear coupling does not occur, so C14=C41=0. A comprehensive comparison is hard to achieve here due to the high discrepancies between the references, e.g., for the B20 structure, one shows a C12 > C44 while the other shows the opposite. In the case of the B2, the tested potential showed a negative C44, while the references showed the same problem as for the B20. Finally, for the B1 structure, one of the references displays a negative C44 and a substantial difference in the bulk modulus.

Our calculations for the Fe-Mo interaction were conducted on both the B1 structure and the reference structure used to build the potential. However, there is a lack of studies in the literature on this system using this structure. It has been observed that Fe-Mo can form a crystal structure with the composition MoFe_2_ belonging to the space group P63/mmc [110]. An earlier study in the literature explored Laves phases, including MoFe_2_ with the configuration [102]. 

The Cr-Si interactions are described to form a base structure that is defined as a P213 cubic structure [103]. As previously noted, we cannot compare our findings for the same quaternary system with any from the literature due to the lack of a fitted potential. In other words, there was no pre-existing mathematical model or framework that could be used to predict the behavior of the Fe-Cr-Si-Mo based on its composition. Some of the relevant previous studies faced similar challenges in developing potentials for novel materials and how they addressed these challenges. For instance, Bonny et al. [111] developed a new potential for a quinary Ni-based alloy (Ni–Fe–Cr–Pd) and compared their results with experimental and theoretical studies. However, due to a lack of the exact quaternary potential, they benchmarked their potential using Ni–Fe–Cr. Similarly, Zhou et al. [112] developed a potential for a quaternary Fe–Ni–Cr–H alloy and validated most of their parameters (e.g., stacking fault energy, diffusion, diffusion energy barrier) against experimental and DFT data because of a lack of established potential for that particular quaternary system. However, there are several studies on the MoSi_2_ compound showing a tetragonal C11b structure [79,106,108]. Our results after the simulation are close to the MEAM reference [79] but deviate from results calculated with DFT and reported experimental measurements [105,106,107,108].

In summary, we can conclude that most of the potential for the binary interactions needs a refitting, as anticipated, due to the merging of all the unary and binary systems. One example of this problem can be shown for the Fe potential. It was strictly fitted for the Fe-Cr interaction, but the Fe-Si and Fe-Mo systems were obtained from another Fe potential, and therefore it needs to be optimized.

In terms of optimized MEAM potential (MEAM-B), during this process, we optimized the MEAM potential parameters. The LAAMPS interatomic potential files are included in the Supplementary Information. Regarding the Cr-Si interaction, a MEAM potential had to be developed from scratch using the DFT results. Table 4 displays a comparison between the optimized MEAM-B potential, the previous unfitted potential, and the references for CrSi. The comparison shows that the optimized MEAM-B potential is in excellent agreement with the DFT calculations. This potential has demonstrated its capability to reproduce the basic structural parameters, which will be incorporated with the rest of the parameters to complete the quaternary system potential. 

A similar approach was adapted to parameterize Mo-Si and Fe-Si binary interactions. Due to a lack of previous studies on Mo-Si, a 1:1 element ratio is adapted from the MoSi2 calculation for elastic and structural properties having a C11b structure [108]. Our optimization generates two final potentials, though fitting displays comparable elastic constants and structural parameters, as seen in Table 5. However, the calculated Ec is lower than DFT/experimental reports. These variations may be attributed to dissimilarities in the computations, fittings, methodologies, and conditions utilized in other studies.

Determining the parameters for the Fe-Si interaction presents a challenge due to the absence of a discernible pattern among the previous literature. Therefore, we investigated two structures with a complete list of elastic and structural parameters. The results are presented in Table 6.

## 3. Results

### 3.1. Bulk Mechanical Properties

In order to obtain the bulk mechanical response of the quaternary alloy, we have used Fe as the base constituent element of the model material and then randomly substituted Fe atoms to reach Febal Cr0.80 Si0.003 Mo0.0096 [16,20,21]. The model of BCC α-Fe with lattice unit of dimension (20 × 20 × 20) containing 16,000 atoms is considered for Cr, Si, and Mo, each with 1257, 61, and 158 atoms relaxed at 300 K, respectively. Bulk modulus B, Young modulus E, shear modulus G, and Poisson’s ratio v were computed using Reuss–Voight equations [114]. The results for our optimized MEAM-B potential are compared with DFT and experimental measurements; see Table 7.

Our calculated properties agree with the previous simulation results but are relatively higher than the reported experimental values. This variance is attributable to several factors, including size effect, defect densities, grain boundaries, material irregularities, and external parameter sets employed in the experimental studies. Keeping these limitations in mind, the computed elastic properties using our MEAM-B potential are reasonably consistent with the experimental values. The relationship between the elastic properties is preserved as the difference between the elastic moduli remains constant, as evidenced by Poisson’s ratio calculated for the ideal crystal.

**Figure 1:**
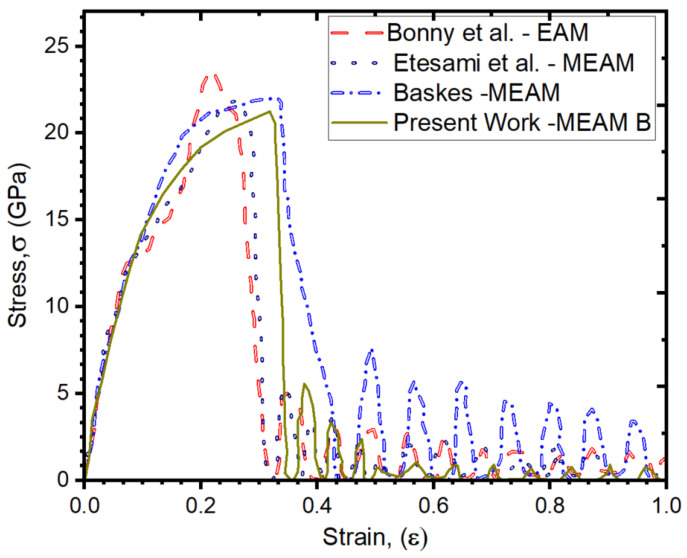
Stress–strain plot for Fe-Fe interaction computed and compared at 300 K [116,117].

Additionally, we plotted stress–strain for the Fe unary system and compared it to the reported results in Figure 1. We performed the stress–strain response calculation at 300 K and a strain rate of 0.001Å/ps, which is consistent with the results documented in the literature. The uniaxial tensile test is performed by applying a uniform strain rate. The calculated results are in good agreement with the previous many-body potentials tested together with our MEAM. Nonetheless, we found that our calculated ultimate tensile strength using the MEAM-B potential was 10% lower than the maximum value reported in the literature, which was approximately 24 GPa [116].

Our results indicate an excellent agreement with previous interatomic potentials.

### 3.2. Our Results Indicate an Excellent Agreement with Previous Interatomic Potentials

The radial distribution function g(r) measures the structural characteristics of alloys and their density as a function of the interatomic distance [83,118]. The probability of locating the position of atoms at a certain radius is mathematically described, where symbols V and Ni represent the volume and number of atoms around a specific atom in the geometry, respectively. The term is the atomic number function representing the ith atom in the system within a radius ranging from r to r+dr, where dr is the radial step [119]. The summation is taken over the number of atoms Ni surrounding the ith atom.

**Figure 2:**
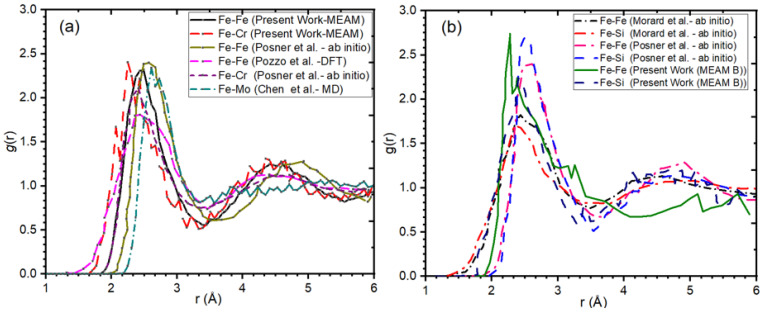
Radial distribution functions for Fe-Cr and Fe-Si pairs: (**a**) Fe-Cr binary pair at 1 bar at 3100 K; (**b**) Fe-Si binary at 1 bar and 3100 K and its comparison with results reported in the literature [77,120,121,122].

We computed the g(r) for Fe-Fe, Fe-Cr, Fe-Si, and Fe-Mo binary pairs for our MEAM potential and those reported in the literature [77,120,121,122]. Figure 2 compares g(r) for Fe-Cr, FeSi, and FeMo pairs at 1 atm pressure at 3100 K for different studies in the literature that were used to parameterize MEAM. The first and second peak for each binary pair lies at ~2.25 Å and ~4.5 Å, consistent with reported values [77,122]. Figure 2a shows the g(r) of the Fe-Cr and Fe-Mo pair with an average density of 9.68 g/cm^3^ and 9.39 g/cm^3^ computed using the proposed MEAM and its comparison with DFT/MD and ab initio results reported earlier [77,120,121,122]. Moreover, Figure 2b represents the RDF of the Fe-Si pair with an average density of 5.92 g/cm^3^ calculated using the proposed MEAM and its comparison with DFT and ab initio results reported earlier [77,120,121,122].

**Figure 3:**
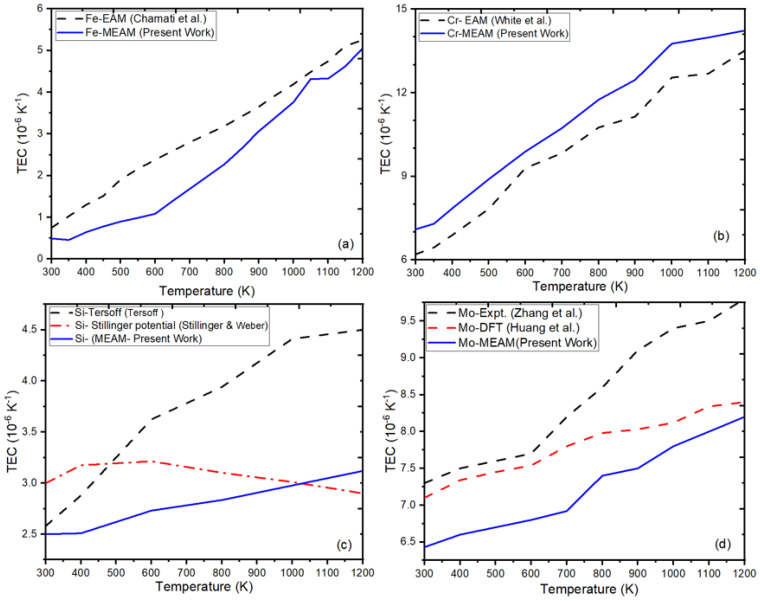
Thermal expansion coefficient (TEC) as a function of temperature for Fe-Cr-Si-Mo: (**a**) TEC of Fe; (**b**) TEC of Cr; (**c**) TEC of Si; and (**d**) TEC of Mo, relative to results reported in previous studies [111,123,125,126].

We used the optimized MEAM-B potential to compute thermal properties compared to the other results reported for unary systems [89,123,124,125,126]. Figure 3a represents the variation of linear thermal expansion percentage as a function of temperature for Fe. The thermal expansion coefficient of our ternary alloy was found to be less than that of pure Fe. Figure 3b–d compare the thermal expansion coefficient of unary elements Cr, Si, and Mo computed using our parameterized MEAM potential and its comparison with existing literature [89,124,125].

**Figure 4:**
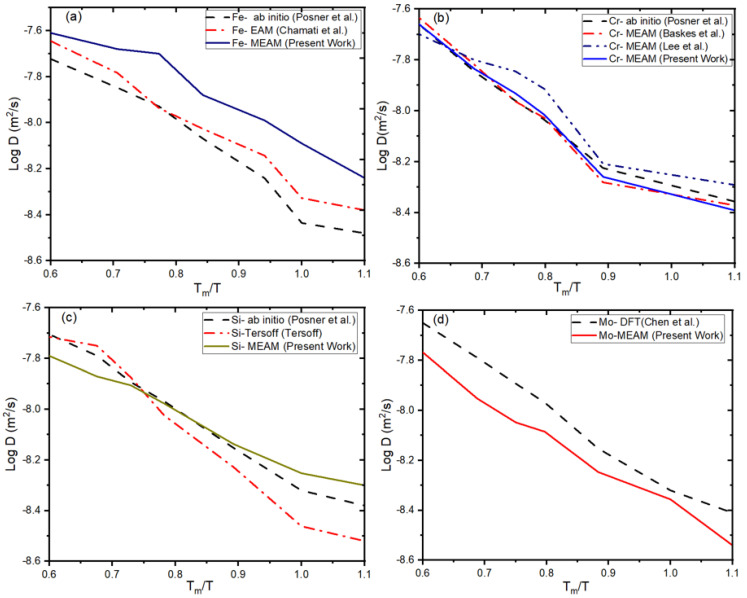
Diffusion coefficient calculations. Diffusion coefficient of (**a**) Fe; (**b**) Cr; (**c**) Si; and (**d**) Mo as a function of homologous temperature calculated using different interatomic potentials in comparison to our MEAM potential [77,79,115,120,123].

Thermal diffusion coefficients were calculated for unary elements simulated at 1 atm pressure as an inverse of homologous temperature, where *T_m_* and *T* are melting and system temperatures, respectively. The self-diffusion coefficient is calculated by the Arrhenius equation, where *D* is the diffusivity constant, *g* is the activation energy, and *T_h_* is the homologous temperature. The self-diffusion coefficients are computed using atomic trajectories in the simulation cell and the asymptotic slope of the time-dependent mean-square displacement [120]. The diffusion coefficient using our optimized MEAM-B potential is in good agreement with other studies [89,93,124]. Figure 4 shows the thermal diffusion coefficient for unary elements reported in the literature and our optimized MEAM. We revealed that values of diffusivity calculated using our optimized MEAM-B potentials around the same temperature ranges are approximately 5.0 × 10^−10^ m^2^/s for Fe, 7.0 × 10^−9^ m^2^/s for Cr, 4.23 × 10^−10^ m^2^/s for Si, and 2.03 × 10^-10^ m^2^/s for Mo, which are consistent with Fe (5.0 × 10^−10^ m^2^/s) [121], Cr (7.7 × 10^−9^ m^2^/s) [77], Si (3.8 × 10^−10^ m^2^/s) [121], and Mo (2.33 × 10^−10^ m^2^/s) [77], respectively. It was seen that thermal diffusion coefficients using our MEAM potential for unary systems are in quite good agreement with each other.

## 4. Conclusions

A MEAM interatomic potential has been created for the Fe-Cr-Si-Mo quaternary system, which is the primary constituent of the T91 alloy. The potential was generated by fitting parameters to unary and binary intermetallic alloys, using DFT and experimental results, through multi-objective optimization (MOO). This development opens possibilities for designing advanced materials, such as MMLCs, for use in nuclear reactors.

The properties calculated using the proposed MEAM potential are in good agreement with the reported values. The mechanical analysis was performed using a tensile test to predict the tensile strength of Fe, and the results were consistent with literature values. The self-diffusion coefficient was also calculated using the Arrhenius diffusion equation near the melting temperature, and the calculated diffusion coefficients for Fe were relatively higher, whereas for Mo, t, they were lower than previously reported. Additionally, the thermal coefficients of all elements, except for Cr, were lower than reported. Nevertheless, our MEAM potential can reliably predict the properties of unary and binary systems, and the calculated properties are consistent with the literature.

This MEAM potential is focused on use for the development of the predictive model of multicomponent alloys (e.g., T91, Fe-Cr-Si, or their combinations) under extreme thermodynamic conditions. The T91 alloy is vital for high-temperature applications, especially advanced fission and fusion reactors. The computational study demonstrates the importance of developing many-body potential for structural materials to evaluate and optimize them under the harsh conditions expected in Generation IV reactors. Moreover, our computed results will append to the existing literature for materials at high temperatures and extreme conditions observed in structural materials.

## Figures and Tables

**Table 1:** Parameters for interactions obtained from each reference.

Method	Reference	System	Systems Extracted
MEAM	Chen et al. [77]	Fe-C-Cr-Mo	Mo
			Fe-Mo
			Cr-Mo
	Choi et al. [81]	Co-Cr-Fe-Mn-Ni	Fe
			Cr
			Fe-Cr
	Jelinek et al. [82]	Al-Si-Mg-Cu-Fe	Si
			Fe-Si
	Baskes [79]	Si-Mo	Si-Mo
DFT	Ren et al. [80]	Cr-Si	Cr-Si

**Table 2:** Unary system’s energetic, structural, and elastic properties for stable structures of Fe, Cr, Si, and Mo as compared to MEAM, DFT, and experimental results. Highlighted rows are our results.

	Cohesive Energy (eV)	Lattice (Å)	Elastic Constants (GPA)			Shear Modulus (GPA)	Young Modulus (GPa)	Bulk Modulus (GPA)	Poisson’s Ratio	Zener’s Ratio (Anisotropic)
	Ec	a_lat_	C_11_	C_12_	C_44_	G	E	B	v	a_r_
**Fe** (bcc)										
MEAM	−4.29	2.863	242.17	138.41	121.46	51.88	141.49	173.00	0.36	2.34
MEAM [77]	−4.29	2.860	302.69	112.78	120.45					
MEAM [81]	−4.29	2.860	226.00	140.00	116.01					
MEAM [93]			243.00	138.00	121.90					
Exp. [95]			243.10	138.10	121.90					
**Cr** (bcc)										
MEAM	−4.10	2.880	344.45	112.89	130.47	115.78	288.71	190.08	0.25	1.13
MEAM [81]			344.50	112.90	130.50					
MEAM [77]	−4.10	2.885	350.00	67.00	100.00					
MEAM [93]			390.90	89.70	103.40					
Exp. [95]			391.00	89.60	103.20					
**Si** (dia)										
MEAM	−4.63	5.431	163.78	64.54	76.46	49.62	127.29	97.62	0.28	1.54
MEAM [96]			164	65	76					
DFT [97]	−4.63	5.429	171.5	67.1	81.1			101.9		
DFT [98]			154.6	58.1	74.4		122.8		0.27	
Exp. [96]			165.8	63.5	79.6					
**Mo** (bcc)										
MEAM	−6.81	3.146	460.18	167.82	110.56	146.18	370.49	265.27	0.27	0.76
MEAM [77]	−6.81	3.149	459.26	167.86	110.79					
MEAM [93]			464.90	165.50	108.80					
Exp. [99]			464.70	161.50	108.90					

**Table 3:** Binary system’s energetic, structural, and elastic properties for MEAM-A potential, where our results are highlighted.

	E_c_	a_lat_	C_11_	C_12_	C_44_	G	E	B	v
Str. Type	eV	Å	(GPa)			GPa	GPa	GPa	
Fe Cr									
B2	−4.31	2.800	348.63	115.85	88.38	116.39	290.84	193.45	0.25
DFT [100]			350.00	150.00	124.00	116.00	295.00	219.00	0.27
Fe Si									
B20	−6.83	4.208	317.42	296.31	13.40	10.56	31.31	303.35	0.48
DFT [82]								226.50	
DFT LDA [100]		4.83	440.00	150.00	190.00			235.00	
DFT [100]		4.48	385.00	120.00	160.00			210.00	
B2	−4.45	2.803	543.04	29.93	−30.40	256.56	539.91	200.97	0.05
DFT [82]					87.00			231.90	
MEAM [82]					36.20			177.70	
DFT LDA [101]		2.70	510.00	160	135.00			285.00	
DFT [101]		2.77	435.00	125	95.00			230.00	
B1	−5.05	4.240	682.15	7.18	52.96	337.48	682.00	232.17	0.01
DFT [82]					−70.00			100.90	
MEAM [82]					65.00			157.90	
Fe Mo									
B1	−4.96	4.702	286.15	109.27	92.77	88.44	225.76	168.23	0.28
MoFe_2_ P63/mmc	−4.81	3.09/7.82 ^†^	64	164	26	−390.86	2818.71	92.01	−0.52
DFT [102]		4.71/7.64 ^†^	441.40	161.50	110.60	126.60		244.90	
Cr Mo									
B2	−4.96	2.901	396.04	204.62	−21.93	95.71	256.64	268.43	0.34
Cr Si									
P213	−9.23	4.550	306.00	316.05	29.85	−5.03	−15.16	312.70	0.51
DFT [103]	−8.13	4.590	390.50	112.30	125.70	130.90	323.80	205.00	0.24
Exp. [104]						107.70	260.90	150	
MoSi									
MoSi_2_ C11b	−5.66	8.80/3.46 ^†^	242.73	156.82	42.31	84.83	s225.37	218.93	0.33
MEAM [79]	−5.92	8.42/3.59 ^†^	252.00	145.00	26.00	75.0	202.00		0.34
DFT [105]	−23.19	7.87/3.22 ^†^						203.70	
DFT [106]		7.79/3.18 ^†^	406.4	111.5	202.1			211.6	
Exp [107]		7.85/3.20 ^†^	410.00	114.90	195.00				
Exp. [108]	19.14 *	2.446	401.00	102.00	208.00			222.0	

† clat/alat (Å). * eV/molecule.

**Table 4:** Comparison of Cr-Si properties before and after the potential fitting. Optimized potential results’ are highlighted.

Cr-Si	E_c_	a_lat_	C_11_	C_12_	C_44_	G	E	B	*v*
P2_1_3	eV	Å	GPa			GPa	GPa	GPa	
MEAM-A	−9.23	4.550	306.00	316.05	29.85	−5.03	−15.16	312.70	0.51
MEAM-B	−7.95	4.601	394.56	112.28	127.34	141.14	344.82	206.37	0.22
DFT [103]	−8.13	4.590	390.50	112.30	125.70	130.90	323.80	205.00	0.24
Exp. [104]						107.70	260.90	150	

**Table 5:** Comparison of Mo-Si_2_ properties for two sets of optimized potentials we obtained, designated by B1 and B2, indicating the second set has a better performance in reproducing MoSi_2_ properties.

MoSi_2_	E_c_	c_lat_/a_lat_	C_11_	C_12_	C_44_	G	E	B	*v*
C11b	eV	Å	GPa			GPa			
MEAM-A	−5.66	8.80/3.46	242.73	156.82	42.31	84.83	225.37	218.93	0.33
MEAM-B1	−5.65	9.55/3.11	396.52	116.16	173.55	158.70	385.09	223.86	0.28
MEAM-B2	−5.645	8.98/3.22	266.36	129.58	178.34	214.12	489.19	227.93	0.24
MEAM [79]	−5.92	8.42/3.59	252.0	145	26.00	75.00	202.00		0.34
DFT [105]	−23.19	7.87/3.22						203.70	
DFT [106]		7.79/3.18	406.4	111.5	202.1			211.6	
Exp. [107]		7.85/3.20	410.0	114.9	195.00				
Exp. [108]	−19.14 *	2.446	401.0	102.00	208.00			222.00	

* eV/molecule.

**Table 6:** Comparison of Fe-Si properties. Results from our optimized potential are highlighted.

FeSi	E_c_	a_lat_	C_11_	C_12_	C_44_	G	E	B	*v*
	eV	Å	GPa			GPa	GPa	GPa	
B20									
MEAM-A	−6.83	4.208	317.42	296.31	13.40	10.56	31.31	303.35	0.48
MEAM-B	−6.41	4.02	328.21	201.23	279.08	12.28	35. 61	298.09	0.45
DFT [82]								226.50	
DFT LDA [101]		4.38	440.00	150	190.00			235.00	
DFT [101]		4.48	385.00	120	160.00			210.00	
Exp. [108]		4.483	345.0	106	138				
B2									
MEAM-A	−4.45	2.803	543.04	29.93	−30.40	256.56	539.91	200.97	0.05
MEAM-B	−4.93	2.63	377.02	146.0	100.0	254.0	548.64	207.57	0.06
DFT [82]					87.00			231.90	
MEAM [82]					36.20			177.70	
DFT LDA [101]		2.70	510.00	160.0	135.00			285.00	
DFT [101]		2.77	435.00	125.0	95.00			230.00	
DFT LDA [113]		2.72	460.0	173.0	114.3			269.5	

**Table 7:** Comparison of computed elastic properties.

	*B* (GPA)	*E* (GPA)	*G* (GPA)	*v*
Fe-Cr-Si-Mo (Present Work)	225	230	86.46	0.33
MEAM Fe-12Cr-2Si [83]	203	228	86.0	0.31
Exp. Fe-12Cr-2Si [115]	157	178	68.0	0.31
Exp. Fe-Cr-Si-Mo [115]	188	192	72.0	0.33

## Data Availability

The raw data required to reproduce these findings are available to download from Supplementary Materials. The processed data required to reproduce these findings are available to download from Supplementary Materials.

## Supplementary Materials

The following supporting information can be downloaded at: https://www.mdpi.com/article/10.3390/ma16072825/s1, including additional figures and tables, and the interatomic potential for LAMMPS. References [76,79,82,83,93,100,101,102,103,104,105,106,107,108,109,127,128,129,130] are cited in the supplementary materials.
