# Supplementary material for: A Modified Embedded-Atom Method Potential for a Quaternary Fe-Cr-Si-Mo Solid Solution Alloy
Source: Materials (Basel). 2023 Apr 1;16(7):2825. doi: 10.3390/ma16072825 (PMC10096159; doi:10.3390/ma16072825)
Supplement: Supplementary file 1 [file materials-16-02825-s001.zip › materials-2293074-supplementary.pdf]

Supplementary Information

Development of Modified Embedded-Atom Potential for Fe-Cr-Si-Mo Quaternary System

Shiddartha Paul[1], Daniel Schwen[2], Michael Short[3], Kasra Momeni[1,*]

[1] *Department of Mechanical Engineering, University of Alabama, Tuscaloosa, AL 35487, US.*

[2]*Department of Computational Mechanics and Materials, Idaho National Laboratory, Idaho Falls, ID 83402, United States.*

[3]*Department of Nuclear Science & Engineering, Massachusetts Institute of Technology, Cambridge, MA 02139, United States.*

[*] Corresponding author's email: kmomeni@ua.edu

1. MEAM potential

Many-body potentials such as MEAM are some of the widely used interatomic potentials of MD simulation. Mathematically

$$V = \sum_i [F(\bar{\rho_i}) + \frac{1}{2}\sum_{j\neq i} S_{ij}\, \emptyset_{ij}(R_{ij})] \qquad \text{(S1)}$$

where the first term is the embedding function of an electron density cloud, and the latter term is pairwise interaction. More details about MEAM potentials can be found in references [1], [2].

2. Fitting of binary potentials

In order to describe each of the binary interactions in the quaternary alloy, the MEAM description requires a definition of many parameters involving individual and multi-element interactions. This method relies on fourteen independent parameters to reproduce the physical properties of the unary system. Three of them—cohesive energy (E_c), nearest neighbor equilibrium distance (r_e), and bulk modulus (B)—were obtained from experimental observations to satisfy the universal equation of state. The rest of the parameters are obtained by fitting them to the values obtained in experimental/DFT results. $\beta^{(0),}$ $\beta^{(1)}$, $\beta^{(2)}$, $\beta^{(3)}$ are the exponential decay factors for the atomic densities, and $t^{(1)}$, $t^{(2)}$, $t^{(3)}$ are weighting factors for the atomic densities, (A) being a scaling factor for the energy-embedding function and for C_{min} and C_{max}, which define the many-body screening parameters. Each of these may have substantial or zero impact depending on the reference

structure, e.g., Table SS1 shows which properties are affected by changing determined parameters for the single bcc elements (adapted from [3]). The plus sign means the effect is significant, the minus sign means the effect is minor, and no sign means no effect.

Regarding the multi-body interactions, the MEAM considers directional bonding described by C_{min} and C_{max}, which represent the limits for the region where the atoms will be screened. In total, we have eight screening parameters depending on their direction. For a binary alloy, the screening can be in four directions *(A, A, B), (B, B, A), (A, B, A),* and *(A, B, B)*, where A and *B* represent the atoms of each element. FigureFigure **S1** shows how these limits interact within the system, taking as a reference the interaction *(A, A, B)*. Atom B is fully unscreened (left, right), and atom B is completely screened (middle).

Table S1. Effect of parameters on individual properties for bcc elements.

	A	$\beta^{(0)}$	$t^{(1)}$	$t^{(2)}$	$t^{(3)}$	C_{min}
C_{11} & C_{12}	+	+		-		+
C_{44}	+	+		-		+
$E_{(surf)}$	+	-	+	-	-	-
E_v^f	+	-	+	+	+	+
$\Delta E_{bcc\rightarrow fcc}$	+	+				+
$\Delta E_{fcc\rightarrow hcp}$	+	+		+		+

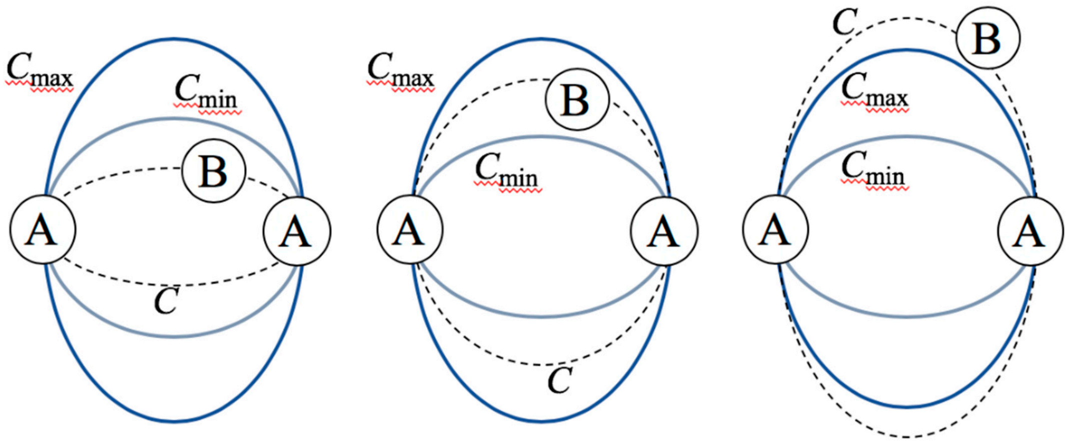

Figure S1. Screening for the interaction (A, A, B).

To describe the total system, we have to define five more parameters: cohesive energy *(E_c),* cutoff radius *(r_c)*, atomic electron density scaling factor (ρ^0) and the relation between bulk modulus, cohesive energy, and the equilibrium atomic volume named alpha (α). In addition, secondary parameters can help us tune our system, such as a scaling factor on the attraction–repulsion interactions. Most of the binary potentials tested were demonstrated to be unable to reproduce DFT and/or experimental parameters fitting. Therefore, proper fitting/refinement is required. The methods used in this investigation for this goal are described in the following sections.

2.1. Multi-Objective Optimization (MOO)

A potential fitting requires the simultaneous optimization of more than one objective function, e.g., to obtain the proper cohesive energy trying to minimize the deviation on the lattice and elastic constants. Following this principle, we adopted a multi-objective optimization procedure as used to develop a potential for magnesium by Houze et al. [4], formulated as min *J(x)* such that $x \in S$, where $J = [J_1(x) \ldots J_m(x)]^T$ and $x = [x_1 \ldots x_n]^T$, being m the objectives that defines the vector J, and the vectors x belonging to the domain S and defining the individual objectives. Given the number of objectives involved in the 2 NN MEAM method, it is unprovable that a set of parameters may optimize all the objectives; therefore, a scalarization with weighting factors was included to minimize all the elements and to avoid the dominance of one objective, implemented as

$$J(x) = \sum_{i=1}^{m} w_i J_i(x) \quad \text{(S2)}$$

The effect of each parameter can be adjusted by w_i. Each objective function is based on the errors between the values obtained and the target reference values. To measure and normalize these differences, we use the expression

$$J_i(x) = \left[\frac{Q_i(x) - Q_i^0}{Q_i^0}\right]^2 \quad \text{(S3)}$$

where Q_i^0 is the reference value and Q_i the value computed by the tested potential. This process was repeated until reaching the minimum error.

2.1.1. Initial parameters

As mentioned, some of the parameters are taken from DFT and/or experimental calculations. These parameters will work as a reference for the upcoming calculations. The values included are cohesive energy, atomic volume, and the bulk modulus, which at the same time can be calculated

from other elemental constants. The equilibrium atomic volume for unary systems is calculated by Eq. SS4, where V represents the volume of the unit cell formed by the lattices, N_o is the number of atoms that forms the unit cell [5], and *c* defines the atomic content of the first element.

$$\Omega = \frac{V}{N_o} \tag{S4}$$

$$\Omega_{Binary} = c\,\Omega_1 + (1-c)\Omega_2 \tag{S5}$$

For binary systems, the equilibrium atomic volume is given by Eq. S5. For the cubic crystal, the elastic properties of bulk modulus are calculated by Voigt–Reuss–Hill approximation [6] for Fe-Si B20, Cr-Mo B2, and Cr-Si $P2_13$ symmetry along axes such that $C_{11}=C_{22}=C_{33}$, $C_{12}=C_{21}=C_{23}=C_{32}=C_{13}=C_{31}$ and $C_{44}=C_{55}=C_{66}$. The off-diagonal shear components are zero, giving $C_{45}=C_{54}=C_{56}=C_{65}=C_{46}=C_{64}=0$, and mixed compression/shear coupling does not occur, therefore $C_{14}=C_{41}=0$ also. Figure S2 **Error! Reference source not found.Error! Reference source not found.Error! Reference source not found.Error! Reference source not found.Error! Reference source not found.Error! Reference source not found.Error! Reference source not found.Error! Reference source not found.**shows the relationship of the elastic constants; on the right-hand side, we can see the matrix for the cubic crystal.

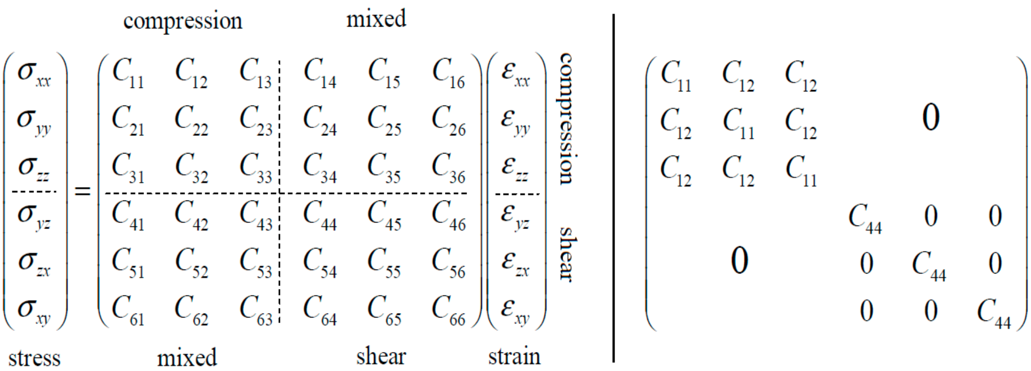

Figure S2. Full range of elastic constants (right) and the matrix reduced for cubic crystals (left).

The bulk modulus for cubic systems is given by

$$B = \frac{C_{11}+2C_{12}}{3}, \tag{S6}$$

and, finally, regardless of the structure, α is calculated using the following equation:

$$\alpha = \left(\frac{9B\Omega}{E_c}\right)^{\frac{1}{2}}. \tag{S7}$$

In this investigation, the binary potentials are already defined except for the Cr-Si potential. Thus, the initial parameters for this interaction are adopted from DFT calculations [7]. The initial/given and calculated parameters are displayed in Table SS2.

Table S2. Initial parameters for the Cr-Si interaction.

Parameter	value
E_c [eV/atom]	-8.13
a [Å]	4.59
Ω_{Si} [Å^3]	20.09
Ω_{Cr} [Å^3]	12.005
Ω_{Total} [Å^3]	16.04
B [eV/ Å]	1.2795
α	4.76

2.1.2. Fitting

The initial parameters set the starting point to develop the potential. We later had to append additional variables, including the eight screening parameters. Other parameters included for purposes of refinement were the attraction and repulsion scaling factors. Two global parameters were not included in the fitting since they would affect the rest of the binary interactions, including r_c and the length of smoothing distance for r_c.

Despite the complex behavior generated by these relationships, Kim and Baskes [8] stated that this method eventually works in many cases. Still, it becomes very tedious and time-consuming given the many combinations and, therefore, potentials to be tested. Consequently, the first step was to identify how heavily these parameters affect the results. The following graphics show how some of these parameters affect the Cr-Si ground state outcomes.

This section will give a full description of the Cr-Si fitting since it was developed from scratch. A refitting follows the same procedure on a minor scale since the limits are defined around the existing parameters. By default, the C_{min} is set to 2.0Å while C_{max} is set to 2.8Å and the attraction=repulsion=0. As a starting point, the C_{min} ranged from 0Å to 2.0Å and the C_{max} from 2.0Å to 2.8Å, whiles the attraction factor ranged from 0 to 1. For the Cr-Si fitting, it was found that the ruling parameters are the C_{min} (A, A, B), C_{min} (B, B, A), C_{max} (A, B, A), and the attraction scaling factor. After defining the behaviors of the different parameters, the strategy was to observe the performance of the intermingled parameters by using a large mesh.

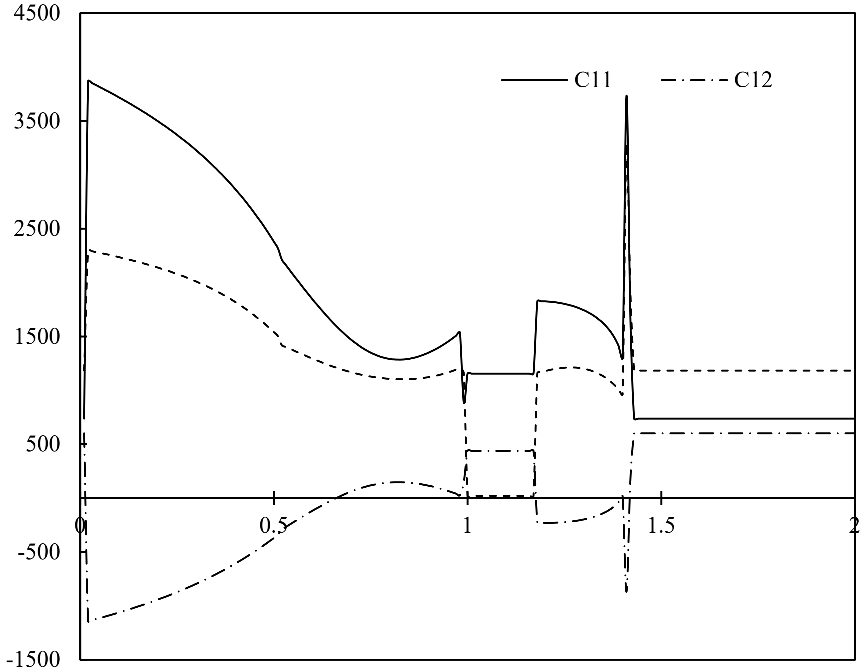

Figure S3. Cr-Si Elastic constants vs. C_{min} (A, A, B).

3.

Table S3. Limits and steps used for the first mesh.

Parameter	Attraction	C_{min} (A, A, B) [Å]	C_{min} (B, B, A) [Å]	C_{max} (A, B, A) [Å]
Limits	0 - 1	0 – 2	0 – 2	2 – 2.8
Step	0.1	0.1	0.1	0.1
Intervals	11	21	21	9
Combination		43659 potentials		

For the optimization, unit cells displaying a *P2₁3* structure were created and relaxed anisotropically at 0K by minimizing the global energy and the total force using the conjugate gradient (CG) method [9]. The boundaries were periodic, allowing the structure lattices to vary during the iterations giving the ground state form for each potential. Ultimately, the system pressure tensors and the structural and energetic parameters were studied. The data were studied and improved using the MOO procedure. Weighting factors were chosen to produce a potential focused on the elastic constants, the ground state energies, and structural parameters, therefore discarding

potentials presenting large discrepancies. This cycle was followed until a minimum error was achieved. As the last step, boxes of different sizes were tested, defining the validity of the potential for the low-energy structures without size limitation.

3. Polycrystalline Calculation

For the bulk polycrystalline material, we needed the full range of elastic constants, i.e., C_{11}, C_{12}, C_{13}, C_{21}, C_{22}, C_{23}, C_{31}, C_{32}, C_{33}, C_{44}, C_{55}, and C_{66}. Then we proceeded to calculate the following properties: the Voigt bulk modulus (B_V) and shear modulus (G_V), defined as

$$9B_v = (C_{11} + C_{22} + C_{33}) + 2(C_{12} + C_{23} + C_{31}) \quad \text{(S8)}$$

$$15G_v = (C_{11} + C_{22} + C_{33}) - (C_{12} + C_{23} + C_{31}) + 3(C_{44} + C_{55} + C_{66}) \quad \text{(S9)}$$

and the Reuss bulk modulus (B_R) and shear modulus (G_R), defined as

$$1/B_R = (S_{11} + S_{22} + S_{33}) + 2(S_{12} + S_{23} + S_{31}), \quad \text{(S10)}$$

$$15/G_R = 4(S_{11} + S_{22} + S_{33}) - 4(S_{12} + S_{23} + S_{31}) + 3(S_{44} + S_{55} + S_{66}), \quad \text{(S11)}$$

where the S_{ij} components come from the elastic flexibility matrix that can be written as the inverse matrix of the elastic stiffness matrix C_{ij}, e.g., $[S_{ij}] = [C_{ij}]^{-1}$. Next, the Hill approximation provides a more realistic representation of the polycrystalline bulk properties, where the average bulk modulus (B) and shear modulus (G) are defined as follows:

$$B = \frac{1}{2}(B_R + B_v) \quad \text{(S12)}$$

$$G = \frac{1}{2}(G_R + G_v) \quad \text{(S13)}$$

Finally, Young's modulus (E) and Poisson's ratio (v) are given by

$$E = \frac{9BG}{3B+G} \quad \text{(S14)}$$

$$v = \frac{3B-2G}{6B+2G} \quad \text{(S15)}$$

The mechanical parameters are computed using these formulas.

4. Supporting files

MEAM.A.meam: The LAMMPS potential file for the developed MEAM-A potential.

MEAM B_Optimized.meam: The LAMMPS potential file for the developed MEAM-B potential.

Library.meam: Library file

References

[1] M. I. Baskes, "Modified embedded-atom potentials for cubic materials and impurities," *Phys. Rev. B*, vol. 46, no. 5, pp. 2727–2742, 1992, doi: 10.1103/PhysRevB.46.2727.

[2] S. Paul, M. Muralles, D. Schwen, M. Short, and K. Momeni, "A Modified Embedded-Atom Potential for Fe-Cr-Si Alloys," *J. Phys. Chem. C*, vol. 125, no. 41, pp. 22863–22871, Oct. 2021, doi: 10.1021/acs.jpcc.1c07021.

[3] M. I. Baskes, B. J. Lee, H. Kim, and Y. Koo Cho, "Second nearest-neighbor modified embedded atom method potentials for bcc transition metals," *Phys. Rev. B - Condens. Matter Mater. Phys.*, vol. 64, no. 18, 2001, doi: 10.1103/PhysRevB.64.184102.

[4] J. Houze *et al.*, "A multi-objective optimization procedure to develop modified-embedded-atom-method potentials: an application to magnesium." pp. 1–8, 2007.

[5] J. William D. Callister, *Materials Science and Engineering 7th Ed. : An Introduction*. 2007.

[6] D. H. Chung and W. R. Buessem, "The Voigt-Reuss-Hill approximation and elastic moduli of polycrystalline Mgo, CaF2, β-ZnS, ZnSe, and CdTe," *J. Appl. Phys.*, vol. 38, no. 6, pp. 2535–2540, 1967, doi: 10.1063/1.1709944.

[7] B. Ren, D.-H. H. Lu, R. Zhou, D.-P. P. Ji, M.-Y. Y. Hu, and J. Feng, "First principles study of stability, mechanical, and electronic properties of chromium silicides," *Chinese Phys. B*, vol. 27, no. 10, p. 107102, 2018, doi: 10.1088/1674-1056/27/10/107102.

[8] S. G. Kim *et al.*, "Semi-empirical potential methods for atomistic simulations of metals and their construction procedures," in *Journal of Engineering Materials and Technology, Transactions of the ASME*, 2009, vol. 131, no. 4, pp. 0412101–0412109, doi: 10.1115/1.3183784.

[9] M. R. Hestenes and E. Stiefel, "Methods of conjugate gradients for solving linear systems," *J. Res. Natl. Bur. Stand. (1934).*, vol. 49, no. 6, p. 409, 1952, doi: 10.6028/jres.049.044.

[10] H. Zhang, G. Wang, M. P. J. Punkkinen, S. Hertzman, B. Johansson, and L. Vitos, "Elastic anomalies in Fe-Cr alloys," *J. Phys. Condens. Matter*, vol. 25, no. 19, 2013, doi: 10.1088/0953-8984/25/19/195501.

[11] B. Jelinek *et al.*, "Modified embedded atom method potential for Al, Si, Mg, Cu, and Fe alloys," *Phys. Rev. B - Condens. Matter Mater. Phys.*, vol. 85, no. 24, 2012, doi: 10.1103/PhysRevB.85.245102.

[12] R. Caracas and R. Wentzcovitch, "Equation of state and elasticity of FeSi," *Geophys. Res. Lett.*, vol. 31, no. 20, pp. 20601–20604, 2004, doi: 10.1029/2004GL020601.

[13] Z. Q. Lv, Z. F. Zhang, Q. Zhang, Z. H. Wang, S. H. Sun, and W. T. Fu, "Structural, electronic and elastic properties of the Laves phases WFe2, MoFe2, WCr2 and MoCr2 from first-principles," *Solid State Sci.*, vol. 56, pp. 16–22, 2016, doi: 10.1016/j.solidstatesciences.2016.03.012.

[14] G. P. Zinoveva, L. P. Andreeva, and P. V Geld, "Elastic constants and dynamics of crystal lattice in monosilicides with B20 structure," *Phys. status solidi*, vol. 23, no. 2, pp. 711–718, 1974, doi: 10.1002/pssa.2210230244.

[15] M. I. Baskes, "Atomistic potentials for the molybdenum-silicon system," *Mater. Sci. Eng. A*, vol. 261, no. 1–2, pp. 165–168, 1999, doi: 10.1016/s0921-5093(98)01062-4.

[16] X. P. Li, S. P. Sun, H. J. Wang, W. N. Lei, Y. Jiang, and D. Q. Yi, "Electronic structure and point defect concentrations of C11b MoSi2 by first-principles calculations," *J. Alloys Compd.*, vol. 605, pp. 45–50, 2014, doi: 10.1016/j.jallcom.2014.03.159.

[17] Z. L. Zhu, H. Z. Fu, J. F. Sun, Y. F. Liu, D. H. Shi, and G. L. Xu, "First-principles calculations of elastic and thermal properties of molybdenum disilicide," *Chinese Phys. Lett.*, vol. 26, no. 8, pp. 1–4, 2009, doi: 10.1088/0256-307X/26/8/086203.

[18] K. Tanaka, H. Onome, H. Inui, M. Yamaguchi, and M. Koiwa, "Single-crystal elastic constants of MoSi2 with the C11b structure," *Mater. Sci. Eng. A*, vol. 239–240, no. 1–2, pp. 188–194, 1997, doi: 10.1016/s0921-5093(97)00580-7.

[19] M. Alouani, R. C. Albers, and M. Methfessel, "Calculated elastic constants and structural properties of Mo and MoSi2," *Phys. Rev. B*, vol. 43, no. 8, pp. 6500–6509, 1991.
